# ﻿Description of two new species of the genus *Trachelas* L. Koch, 1872 and the male of *T.gaoligongensis* Jin, Yin & Zhang, 2017 from China (Araneae, Trachelidae)

**DOI:** 10.3897/zookeys.1215.130564

**Published:** 2024-10-14

**Authors:** Gang Tang, Wen-long Yan, Yi Zhao, Xian-jin Peng

**Affiliations:** 1 College of Life Sciences, Hunan Normal University, Changsha, Hunan 410081, China Hunan Normal University Changsha China

**Keywords:** Morphology, spider, taxonomy, trachelids, Yunnan

## Abstract

Two new spider species of the genus *Trachelas* L. Koch, 1872 are described from China: *Trachelaskavanaughi***sp. nov.** (♀) and *Trachelasventriosus***sp. nov.** (♀). The male of *Trachelasgaoligongensis* Jin, Yin & Zhang, 2017 is described for the first time. Illustrations of the body and copulatory organs and a distribution map are provided.

## ﻿Introduction

The subfamily Tracheleae Simon, 1897 was originally treated as a member of the family Corinnidae Karsch, 1880. Ramírez (2014) separated it from Corinnidae and elevated it to its own family, Trachelidae. This family currently contains 290 species in 25 genera, of which nine genera and 38 species are distributed in China. *Trachelas* L. Koch, 1872 is the most species-rich genus in Trachelidae, with 91 species distributed worldwide, including 13 species in China (mainly distributed in southwest China) (WSC 2024). There have been seven new species of *Trachelas* described and one new record reported in China recently (Zhang et al. 2009; Jin et al. 2017; Liu et al. 2024).

During the examination of spider specimens collected from Yunnan Province in 2007, two new species, *Trachelasventriosus* sp. nov. (♀), *T.kavanaughi* sp. nov. (♀), and the males of *T.gaoligongensis* Jin, Yin & Zhang, 2017 were discovered. Descriptions and photomicrographs of the habitus and copulatory organs and distribution map are provided.

## ﻿Material and methods

Specimens were stored in 75% ethanol. The female genitalia were cleared with lactic acid before examination and photography. Specimens were photographed using a Kuy Nice E3IS PM digital camera attached to an Olympus BX53 compound microscope and examined and measured with a Leica M205C stereomicroscope. Photographs were taken by placing specimens on alcohol-soaked cotton in a Petri dish. Focus-stacked images were composited using Helicon Focus ver. 7.0 and then modified in Adobe Photoshop CS6. All measurements are in millimeters (mm). Leg measurements are as follows: total length (femur, patella+tibia, metatarsus, tarsus). All specimens are deposited at the College of Life Sciences, Hunan Normal University (**HNU**), Changsha, Hunan Province, China.

The following abbreviations are used in the text and figures: **ALE** anterior lateral eyes, **AME** anterior median eyes, **ATR** atrium, **CD** copulatory duct, **CnD** connecting duct, **CO** copulatory opening, **RTA** retrolateral tibial apophysis, **E** embolus, **FD** fertilization duct, **MOA** median ocular area, **PLE** posterior lateral eyes, **PME** posterior median eyes, **RPA** retrolateral patellar apophysis, **SD** sperm duct, **ST** subtegulum, **ST1** primary spermatheca, **ST2** secondary spermatheca, **TA** tegular apophysis, **VFG** ventral femoral groove.

## ﻿Taxonomy

**Family Trachelidae Simon, 1897**

### ﻿Genus Trachelas

Taxon classificationAnimaliaAraneaeTrachelidae

L. Koch, 1872

947A3FBF-EC79-5E77-8CAE-AC9F9302FBD1

#### Type species.

*Trachelasminor* O. Pickard-Cambridge, 1872.

### ﻿ Trachelas gaoligongensis

Taxon classificationAnimaliaAraneaeTrachelidae

Jin, Yin & Zhang, 2017

DA69F347-BAE3-5AC5-ABD3-29AA1532EFC2

Figs 1 , 2 , 3 , 4

Trachelas gaoligongensis Jin, Yin & Zhang, 2017: 42, figs 16A−G, 18A, B (♀).

#### Material examined.

• 4 ♂, 1 ♀(HNU-20071010); China, Yunnan Prov., Longyang County, Mangkuan Baihualing; 25.30366°N, 98.80032°E; 1624 m a.s.l.; 10 October 2007; Xian-jin Peng leg. • 2 ♀ (HNU-Tang-04-12); China, Yunnan Prov., Gongshan County, Cikai Township, Heiwadi Village; 27.47101°N, 98.35533°E; 1850 m a.s.l.; 13−16 November. 2004; Guo Tang leg. • 2 ♀ (HNU-DHK-2004-082); China, Yunnan Prov., Gongshan County, Dulongjiang Township, 0.2 km S of confluence of Dulongjiang with Muke Wang [river]; 27.84125°N, 98.32979°E; 1450 m a.s.l.; 11 November 2004; D. H. Kavanaugh leg. • 1 ♀ (HNU-VFL-04-0027); China, Yunnan Prov., Gongshan County, Bingzhongluo Township, west side of bridge NW of Stone Gate; 28.06670°N, 98.58890°E; 1500 m a.s.l.; 11 December 2004; V. F. Lee leg.

#### Etymology.

The species name “gaoligongensis” refers to the Gaoligong mountain range where the type locality is found, adjective.

#### Diagnosis.

The male of *Trachelasgaoligongensis* (Figs 2, 4A−C) resembles that of *T.bomiensis* Jin & Mi, 2024 (see Liu et al. 2024, fig. 2A−D) in having a hook-shaped sperm duct and a protruding genital bulb but differs as follows: (1) the embolus is enlarged at the base and elongated at the tip in retrolateral view (vs. elongated at the base and with two spirals at the tip); (2) the retrolateral tibial apophysis points to the dorsal side of the cymbium in prolateral view (vs. absent); and (3) the retrolateral patellar apophysis is longitudinally bar-shaped, distally covered with feathery setae in retrolateral view (vs. distal portion transversely bent toward tibia and without feathery setae). The female of *T.gaoligongensis* (Figs 3, 4D, E) resembles that of *T.kavanaughi* sp. nov. (see Figs 5E, F, 7A, B) in the shape of the atrium and secondary spermathecae, but differs as follows: (1) the atrium is about as long as wide in ventral view (vs. wider than long); (2) the copulatory openings are posteriorly located on the genitalia in ventral view (vs. anteriorly located on the genitalia); (3) the primary spermathecae are oval in dorsal view (vs. round); and (4) the interdistance of the secondary spermathecae is more than twice the width of the primary spermathecae in dorsal view (vs. narrower than the width of the primary spermathecae).

#### Description.

**Figure 1.**
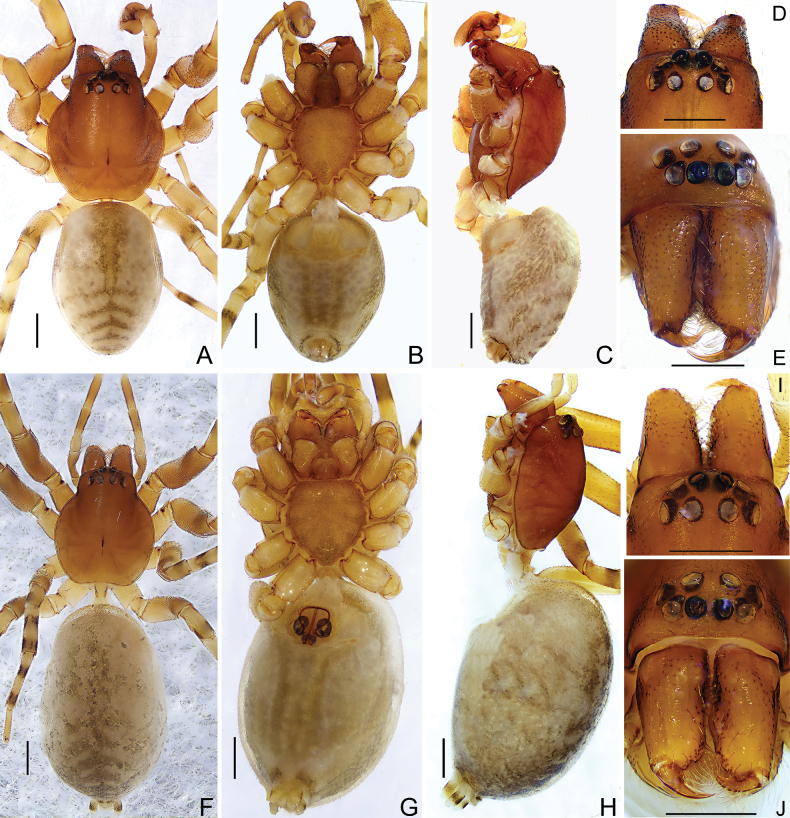
*Trachelasgaoligongensis* Jin, Yin & Zhang, 2017 (HNU-20071010). Male (**A−E**) **A** habitus, dorsal view **B** ditto, ventral view **C** ditto, lateral view **D** ocular area, dorsal view **E** carapace, frontal view. Female (**F−J**) **F** habitus, dorsal view **G** ditto, ventral view **H** ditto, lateral view **I** ocular area, dorsal view **J** carapace, frontal view. Scale bars: 0.5 mm.

**Male.** (one of HNU-20071010) (Fig. 1A−E). Total length 4.06. Carapace 2.05 long, 1.64 wide; abdomen 2.30 long, 1.65 wide. Carapace brown, fovea thin and black, cervicle and radial grooves distinct. Chelicerae and labium brown, three promarginal and two retromarginal teeth. Sternum and endites yellowish brown, truncated margin of sternum with distinct crescent-shaped depression. Eye sizes and interdistances: AME 0.14, ALE 0.14, PME 0.14, PLE 0.11, AME−AME 0.06, AME−ALE 0.02, PME−PME 0.11, PME−PLE 0.10, ALE−PLE 0.08. MOA 0.34 long, anterior width 0.28, posterior width 0.36. Clypeus height 0.12. Legs yellowish brown, with black rings. Leg measurements: leg I 5.38 (1.68, 2.19, 0.92, 0.59), II 5.24 (1.61, 2.13, 0.87, 0.63), III 3.15 (0.87, 0.90, 0.94, 0.44), IV 5.60 (1.74, 1.83, 1.45, 0.58). Leg formula: 4123. Abdomen oval, apricot-white; anterior half of dorsum with two black-brown longitudinal stripes; posterior half with five black-brown chevrons decreasing in size from anterior to posterior; venter with three dark longitudinal stripes. Spinnerets with parenthesis-shaped marks laterally.

**Figure 2.**
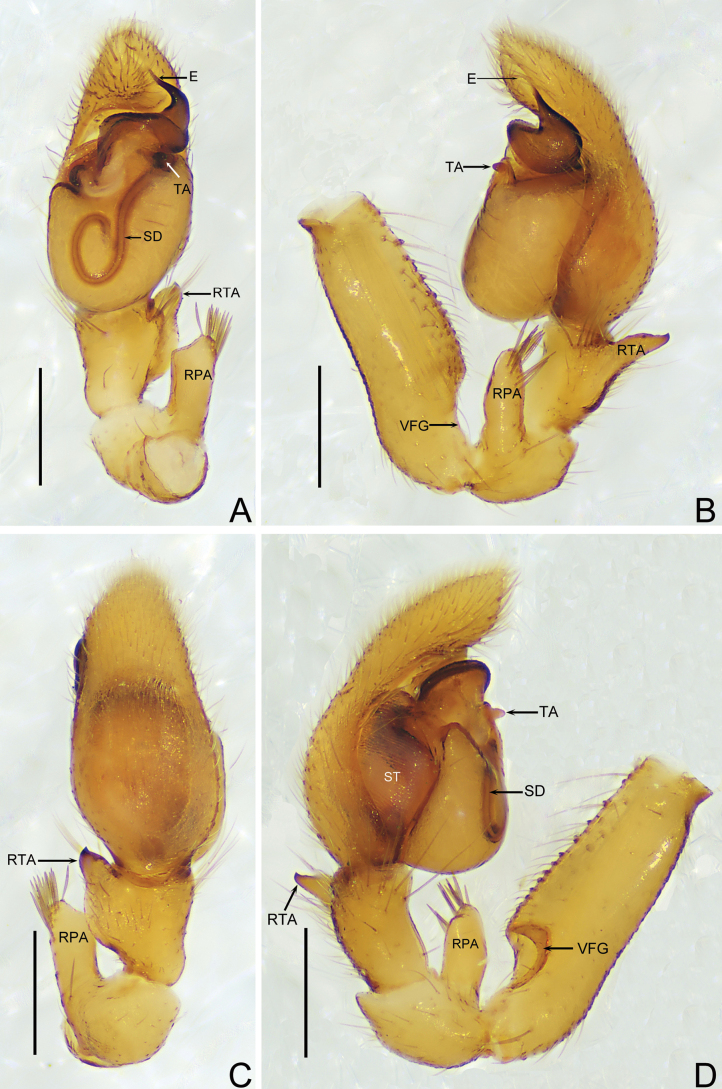
*Trachelasgaoligongensis* Jin, Yin & Zhang, 2017 (HNU-20071010) **A** male left palp, ventral view **B** ditto, retrolateral view **C** ditto, dorsal view **D** ditto, prolateral view. Scale bars: 0.3 mm.

Palp (Figs 2, 4A−C). Retrolateral patellar apophysis finger-like, as long as patella, distally covered with feathery setae; retrolateral tibial apophysis spur-like, as long as tibia, pointed distally; genital bulb oval, embolus short, with base broad and spiralled, apex constricted and spinelike. Sperm duct distinct and hook-shaped.

**Female.** (HNU-20071010) (Fig. 1F−J). Total length 4.95. Carapace 1.70 long, 1.39 wide; abdomen 2.84 long, 1.79 wide. Eye sizes and interdistances: AME 0.09, ALE 0.09, PME 0.11, PLE 0.11, AME−AME 0.08, AME−ALE 0.02, PME−PME 0.10, PME−PLE 0.09, ALE−PLE 0.08. MOA 0.28 long, anterior width 0.24, posterior width 0.32. Clypeus height 0.11. Leg measurements: leg I 4.81(1.50, 1.80, 0.89, 0.62), II 4.57 (1.44, 1.63, 0.91 0.59), III 3.73 (1.19, 1.12, 0.91, 0.51), IV 5.47 (1.54, 1.91, 1.40, 0.62). Leg formula: 4123. Abdomen oblong, anterior half of dorsum with a black-brown longitudinal stripe and some irregular darker patches. Other characters as in male.

**Figure 3.**
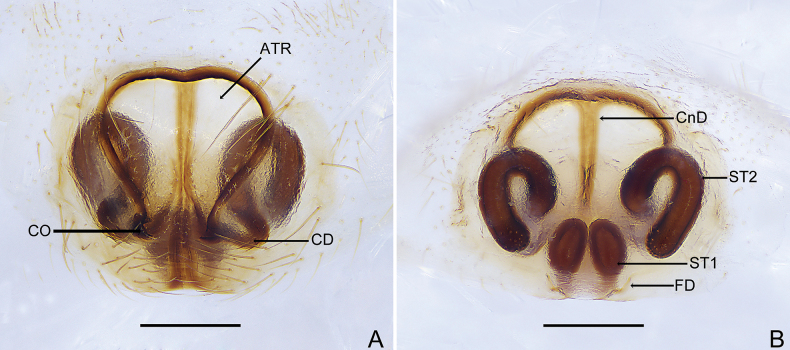
*Trachelasgaoligongensis* Jin, Yin & Zhang, 2017 (HNU-20071010) **A** genitalia, ventral view **B** ditto, dorsal view. Scale bars: 0.3 mm.

**Figure 4.**
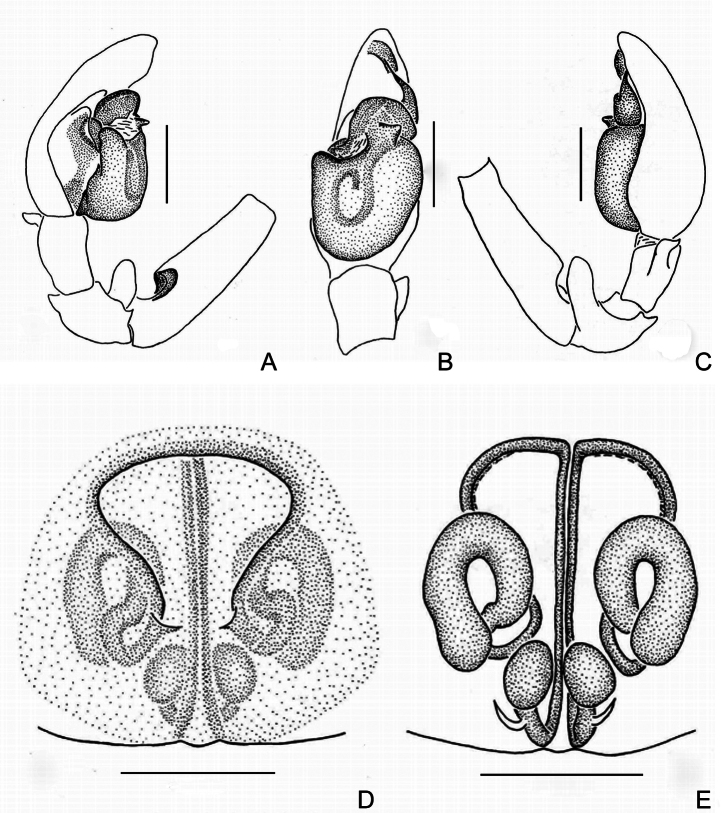
*Trachelasgaoligongensis* Jin, Yin & Zhang, 2017 **A** male left palp, prolateral view **B** ditto, ventral view **C** ditto, retrolateral view **D** genitalia, ventral view **E** ditto, dorsal view. Scale bars: 0.3 mm.

Epigyne (Figs 3, 4D, E). Atrium about as long as wide, copulatory openings pore-like, located at posterior of epigyne; copulatory ducts C-shaped, connected to n-shaped secondary spermathecae; connecting ducts slender, axisymmetric; primary spermathecae oval, connected to lightly sclerotized fertilization ducts.

#### Distribution.

China (Yunnan) (Fig. 8).

### ﻿ Trachelas kavanaughi sp. nov.

Taxon classificationAnimaliaAraneaeTrachelidae

F97917EB-8670-5AF3-9C18-CF2B37A5C6CC

https://zoobank.org/6250BA11-335B-45DD-B0BB-F65CCE47FF41

Figs 5 , 7A, B

#### Type material.

***Holotype*** • ♀ (HNU-DHK-2004-058); China, Yunnan Prov., Gongshan County, Dulongjiang Township, south of Dizhengdang Village along Silalong River; 28.07654°N, 98.32603°E; 1890 m a.s.l.; 28 October 2004; D. H. Kavanaugh leg.

#### Etymology.

The species is named in honor of the type specimen collector, D. H. Kavanaugh, the curator emeritus at the California Academy of Sciences.

#### Diagnosis.

**Figure 5.**
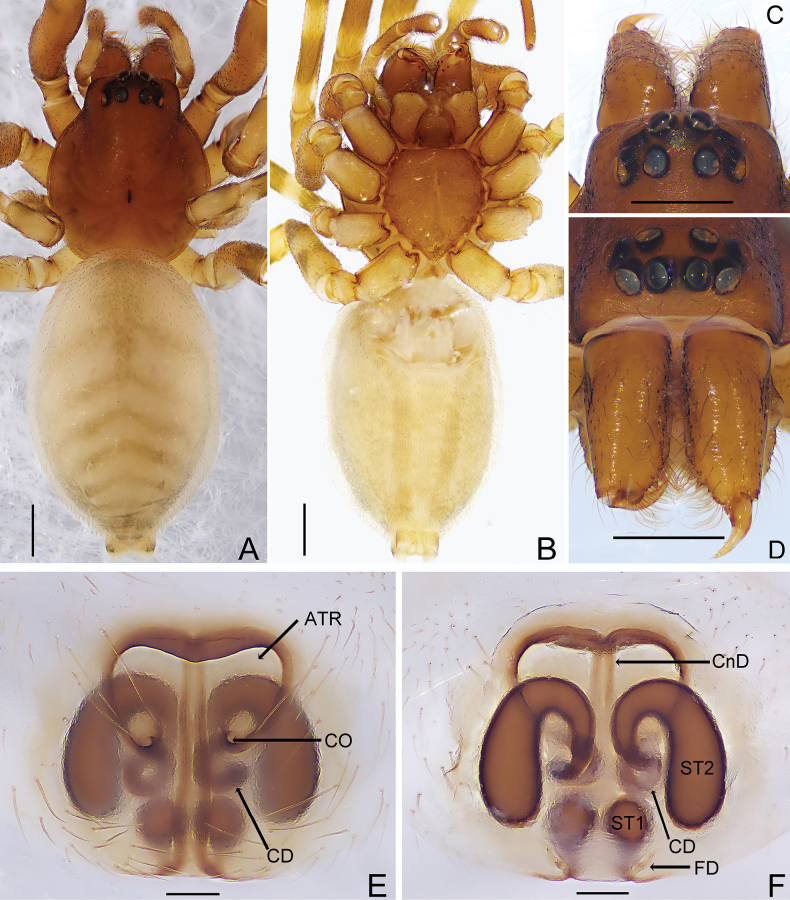
*Trachelaskavanaughi* sp. nov., female (holotype) **A** habitus, dorsal view **B** ditto, ventral view **C** ocular area, dorsal view **D** carapace, frontal view **E** genitalia, ventral view **F** ditto, dorsal view. Scale bars: 0.5 mm (**A−D**); 0.3 mm (**E, F**).

The female of this new species (Figs 5E, F, 7A, B) resembles that of *Trachelasgaoligongensis* (see Figs 3, 4D, E) in the shape of the atrium and the secondary spermathecae but differs as follows: (1) the atrium is wider than long in ventral view (vs. about as long as wide); (2) the copulatory openings are located on the anterior of the genitalia in ventral view (vs. located on posterior); (3) the primary spermathecae are round in dorsal view (vs. oval); and (4) the interdistance of the secondary spermathecae is narrower than the width of the primary spermathecae in dorsal view (vs. more than the twice width of the primary spermathecae).

#### Description.

**Female** (holotype) (Fig. 5A−D). Total length 4.45. Carapace 1.75 long, 1.61 wide; abdomen 2.68 long, 1.79 wide. Carapace brown, smooth, fovea black, small and distinct. Chelicerae brown, with three promarginal and two retromarginal teeth. Sternum and labium light brown, and truncated margin of sternum with distinct crescent-shaped depression. Eye sizes and interdistances: AME 0.13, ALE 0.13, PME 0.13, PLE 0.13, AME−AME 0.05, AME−ALE 0.02, PME−PME 0.10, PME−PLE 0.08, ALE−PLE 0.05. MOA 0.27 long, anterior width 0.30, posterior width 0.34. Clypeus height 0.12. Legs light brown alternating with dark brown. Leg measurements: leg I 5.53 (1.72, 2.02, 1.04, 0.75), II 5.30 (1.60, 1.93, 1.06, 0.71), III 4.20 (1.22, 1.43, 1.02, 0.53), IV 5.72 (1.66, 1.95, 1.46, 0.65). Leg formula: 4123. Abdomen oval, apricot-white; dorsum with five black-brown chevrons decreasing in size from anterior to posterior; venter with two blurry gray longitudinal stripes. Spinnerets yellowish.

Epigyne (Figs 5E, F, 7A, B). Atrium wider than long copulatory openings small, located at anterior of epigyne; copulatory ducts C-shaped, secondary spermathecae narrowest at junction with copulatory ducts, widening from copulatory openings to spermathecae; connecting ducts axisymmetric; primary spermathecae round, connected to lightly sclerotized fertilization ducts.

**Male.** Unknown.

#### Distribution.

Known only from the type locality (Fig. 8).

### ﻿ Trachelas ventriosus sp. nov.

Taxon classificationAnimaliaAraneaeTrachelidae

49B78E18-C507-5F42-B7C3-989C69AD6E4A

https://zoobank.org/87DF29C7-02FA-411D-8A54-CBC9141565F7

Figs 6 , 7C, D

#### Type material.

***Holotype*** • ♀ (HNU-Wang060528-1); China, Yunnan Prov., Tengchong County, Houqiao Township; 25.35391°N, 98.25488°E; 1785 m a.s.l.; 28 May 2006; Xin-Ping Wang, Peng Hu leg.

#### Etymology.

The species name is derived from the Latin “ventriosus” (pot-bellied), referring to its large abdomen; adjective.

#### Diagnosis.

**Figure 6.**
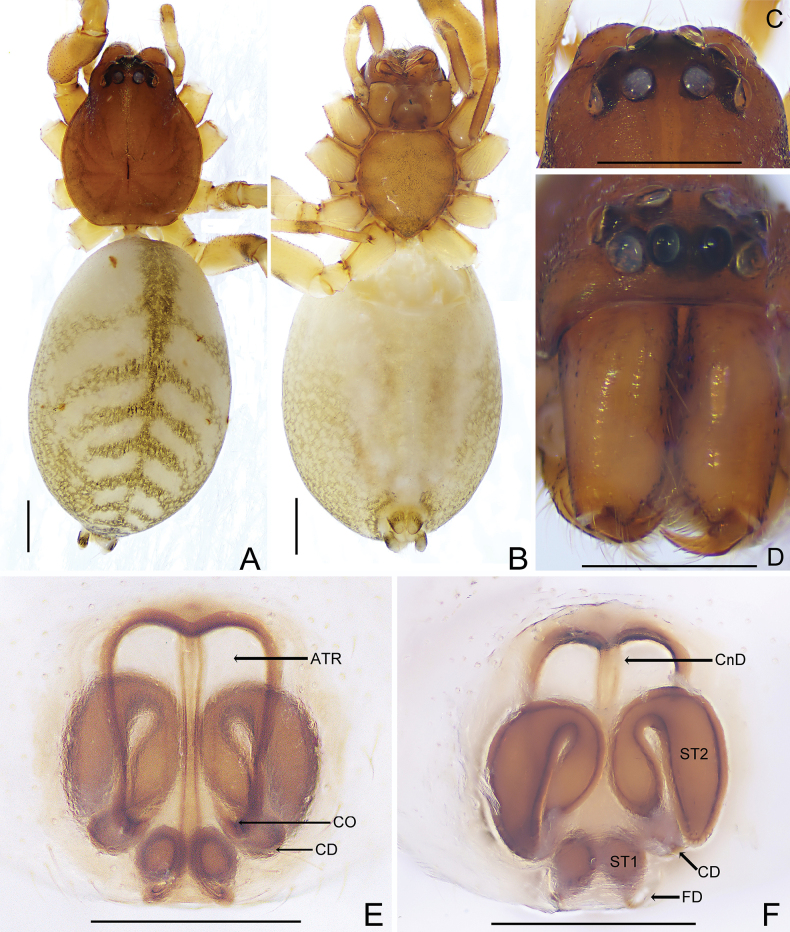
*Trachelasventriosus* sp. nov., female (holotype) **A** habitus, dorsal view **B** ditto, ventral view **C** ocular area, dorsal view **D** carapace, frontal view **E** genitalia, ventral view **F** ditto, dorsal view. Scale bars: 0.5 mm (**A−D**); 0.3 mm (**E, F**).

**Figure 7.**
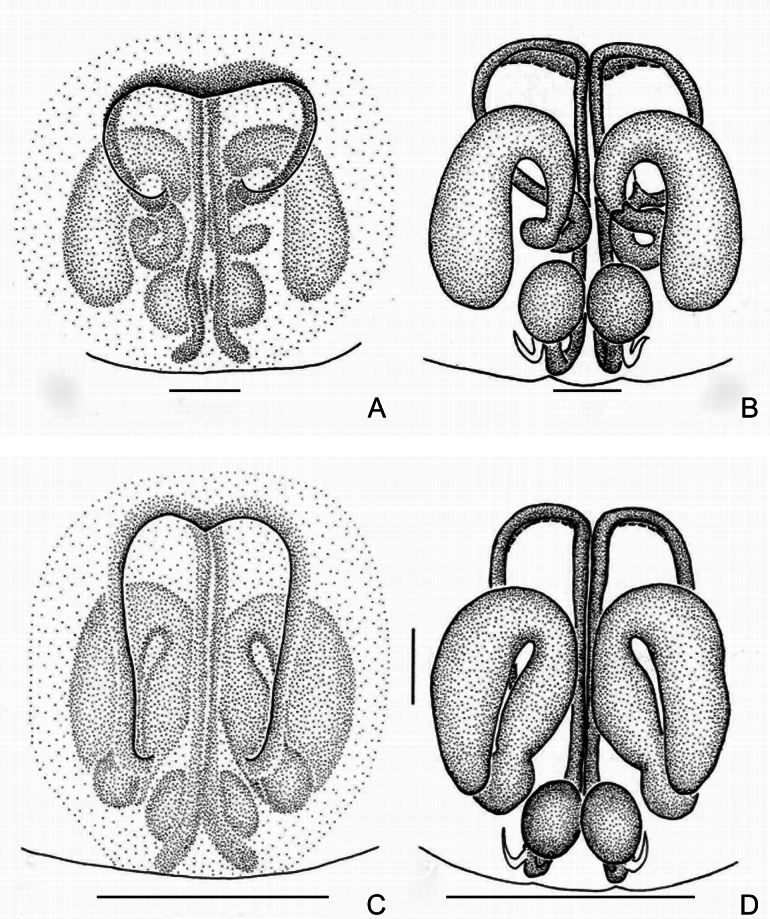
**A, B***Trachelaskavanaughi* sp. nov. **C, D***Trachelasventriosus* sp. nov. **A** genitalia, ventral view **B** ditto, dorsal view **C** ditto, ventral view **D** ditto, dorsal view. Scale bars: 0.3 mm.

The female of this new species (Figs 6E, F, 7C, D) resembles that of *Trachelasfasciae* Zhang, Fu & Zhu, 2009 (see Zhang et al. 2009, figs 21, 22) in having symmetrical connecting ducts and the primary spermathecae are near the genital groove but differs as follows: (1) the atrium occupies 3/4 of the genitalia in ventral view (vs. 1/3 of the genitalia); (2) the copulatory openings are posterior to the secondary spermathecae in ventral view (vs. anterior to the secondary spermathecae); (3) the secondary spermathecae are inverted V-shaped in dorsal view (vs. V-shaped); and (4) the primary and secondary spermathecae are far away from each other in dorsal view (vs. partially overlapping).

#### Description.

Female (holotype) (Fig. 6A−D). Total length 4.47. Carapace 1.59 long, 1.33 wide; abdomen 2.87 long, 1.89 wide. Carapace brown, fovea black and slender, radial grooves distinct. Chelicerae light brown, with two promarginal and three retromarginal teeth. Sternum and labium light brown, partly covered with black setae. Eye sizes and interdistances: AME 0.12, ALE 0.12, PME 0.12, PLE 0.12, AME−AME 0.06, AME−ALE 0.01, PME−PME 0.10, PME−PLE 0.08, ALE−PLE 0.07. MOA 0.26 long, anterior width 0.24, posterior width 0.30. Clypeus height 0.11. Legs light brown alternating with dark brown. Leg measurements: leg I 4.56 (1.43, 1.65, 0.81, 0.67), II 4.82 (1.44, 1.62, 1.22, 0.54), III 3.02 (0.83, 1.05, 0.72, 0.42), IV 4.80 (1.32, 1.71, 1.23, 0.54). Leg formula: 2413. Abdomen oval, apricot-white; dorsum with eight black-brown chevrons decreasing in size from anterior to posterior, with a longitudinal black-brown stripe in middle, and several brown markings distributed irregularly; venter with two blurry gray longitudinal stripes. Spinnerets covered with black setae and parenthesis-shaped marks laterally.

Epigyne (Figs 6E, F, 7C, D). Atrium longer than wide, copulatory openings located on posterior of epigyne; connecting ducts long and symmetrical; secondary spermathecae close to each other; primary spermathecae close to genital groove, connected by short fertilization ducts.

**Male.** Unknown.

#### Distribution.

**Figure 8.**
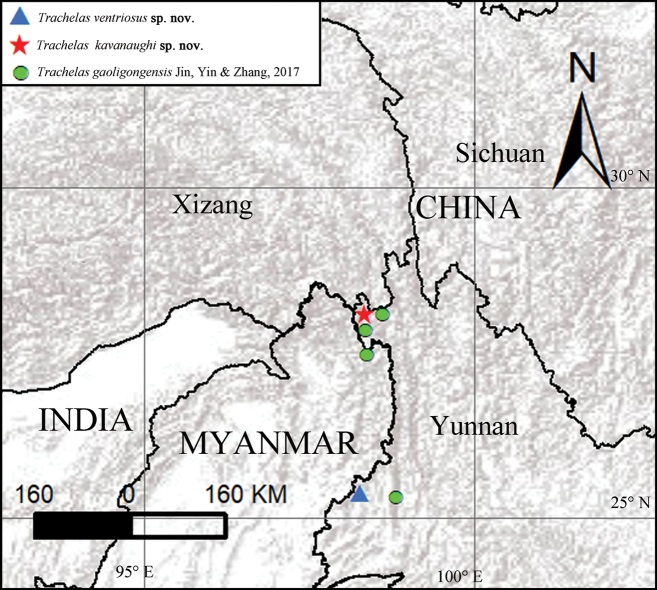
Collection localities of *Trachelasgaoligongensis* Jin, Yin & Zhang, 2017, *Trachelaskavanaughi* sp. nov. and *Trachelasventriosus* sp. nov.

Known only from the type locality (Fig. 8).

## Supplementary Material

XML Treatment for Trachelas

XML Treatment for Trachelas gaoligongensis

XML Treatment for Trachelas kavanaughi

XML Treatment for Trachelas ventriosus

## ﻿Additional information

### Author ORCIDs

Gang Tang https://orcid.org/0009-0009-6287-6706

Wen-long Yan https://orcid.org/0009-0001-9663-9436

Yi Zhao https://orcid.org/0009-0008-8804-5305

Xian-jin Peng https://orcid.org/0000-0002-2614-3910

### Data availability

All of the data that support the findings of this study are available in the main text.
